# Supplementary material for: Systematic reduction of hyperspectral images for high-throughput plastic characterization
Source: Sci Rep. 2023 Dec 7;13:21591. doi: 10.1038/s41598-023-49051-y (PMC10703868; doi:10.1038/s41598-023-49051-y)
Supplement: Supplementary file 1 — Supplementary Information. [file 41598_2023_49051_MOESM1_ESM.docx]

**Systematic reduction of Hyperspectral Images for high-throughput Plastic Characterization**

*Mahdiyeh Ghaffari^1^*, Mickey C. J. Lukkien^1^, Nematollah Omidikia^1^, Gerjen H. Tinnevelt^1^, Marcel C. P. van Eijk^2^, Stanislav Podchezertsev^2^, Jeroen J. Jansen^1*^*

*^1^Radboud University, Institute for Molecules and Materials, Analytical Chemistry, P.O. Box 9010, 6500 GL Nijmegen, the Netherlands*

*^2^National Test Centre Circular Plastics (NTCP), Duitslanddreef 7, 8447 SE Heerenveen, the Netherlands*

*Email:* [*mahdiyeh.ghaffari@ru.nl*](mailto:mahdiyeh.ghaffari@ru.nl)*,* [*chemometrics@ru.nl*](mailto:chemometrics@ru.nl)

**Supporting Information**

This paper utilizes correlation growing for object detection, where the algorithm performs semantic segmentation in a hyperspectral image. This approach resembles how a flood-fill algorithm in a photoshopping program can fill an enclosed area with a particular color. Although the algorithm has not been published as an independent research paper yet, it functions by selecting a seed pixel arbitrarily, which belongs to an object. The algorithm then compares the corresponding spectra of the seed to its neighboring pixels by computing the correlation coefficient. The pixels with a high/low correlation are identified as part of the object/empty conveyor belt, respectively. The seed continues to expand by checking the neighboring pixels of newly recognized object pixels. Once there are no additional pixels with adequate correlation, the algorithm stops, indicating that the edge of the object has been reached. This process is illustrated in Figure S1b for an arbitrary object (Figure S1a).

In the context of plastic sorting, where real-time prediction and classification are of paramount importance, it's essential to recognize that the landscape accommodates full image analysis. This can involve a combination of line scans to construct an image or the utilization of actual 2D images, a practice well-established in applications involving both RGB and HSI data. It is noteworthy that manufacturers often maintain a degree of secrecy regarding the intricate workings of their equipment, underscoring the significance of data interpretation and treatment. Furthermore, the transition between line scans and 2D scans can be effectively facilitated through appropriate data manipulation and interpretation techniques, with the choice between these approaches being contingent upon specific sorting requirements and available data.

| **a**  **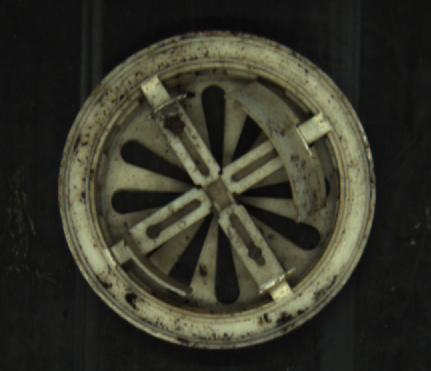** | **b**  **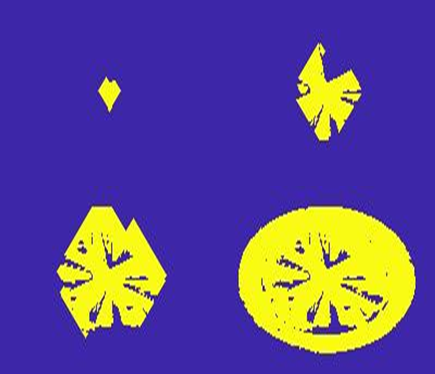** |
| --- | --- |

**Figure S1**. a) Present the RGB image of an arbitrary object and b) is visualizing the object

detection using correlation growing.

| **a**  **** | **b**  **** | **c**  **** |
| --- | --- | --- |
| **d**  **** | **e**  **** | **f**  **** |

**Figure S2.** a) The mean image of the first experimental case is visualized. b-f) Present the classification results of the first case study. The black dots are results of prediction.

| **a**  **** | **b**  **** | **c**  **** |
| --- | --- | --- |
| **d**  **** | **e**  **** | **f**  **** |

**Figure S3.** a) The mean image of the second experimental case is visualized. b-f) Present the classification results of the second case study. The black dots are results of prediction. Object 2 and 3, which are both multilayers, are very overlapped in the score plots.

The proposed strategy in this contribution builds upon the prior findings, introducing the concept of selecting essential spectral pixels and spatial variables to reduce redundancy and enhance data analysis. Extending this foundation, by introducing a novel object detection method employing a correlation-growing algorithm. This approach effectively uncovers essential information about each object, demonstrating adaptability and utility in various scenarios, including multicomponent and multilayer packaging materials.

Furthermore, while modern optical sorters excel at identifying mono-material packaging, our work introduces a pioneering method that swiftly identifies the material composition of intricate multilayered structures commonly found in flexible packaging. This breakthrough represents a significant advancement, addressing a crucial need in the recycling industry. We recognize the time constraints in real-time waste sorting operations, where conveyor belt speeds often reach 2 to 3 meters per second, leaving less than 160 milliseconds for detection and sorting decisions. In contrast to traditional approaches that rely on spectral comparisons to a database, our method is notably faster and more efficient. This efficiency enables comprehensive analysis before sorting, a valuable feature, especially in handling complex multilayered structures. This clarification highlights the innovation in this work and its potential to enhance the efficiency and accuracy of plastic sorting, particularly for multilayered plastics.
